# Getting to NO Alzheimer's Disease: Neuroprotection versus Neurotoxicity Mediated by Nitric Oxide

**DOI:** 10.1155/2016/3806157

**Published:** 2015-11-30

**Authors:** Rachelle Balez, Lezanne Ooi

**Affiliations:** Illawarra Health and Medical Research Institute, School of Biological Sciences, University of Wollongong, Wollongong, NSW 2522, Australia

## Abstract

Alzheimer's disease (AD) is a neurodegenerative disorder involving the loss of neurons in the brain which leads to progressive memory loss and behavioral changes. To date, there are only limited medications for AD and no known cure. Nitric oxide (NO) has long been considered part of the neurotoxic insult caused by neuroinflammation in the Alzheimer's brain. However, focusing on early developments, prior to the appearance of cognitive symptoms, is changing that perception. This has highlighted a compensatory, neuroprotective role for NO that protects synapses by increasing neuronal excitability. A potential mechanism for augmentation of excitability by NO is via modulation of voltage-gated potassium channel activity (Kv7 and Kv2). Identification of the ionic mechanisms and signaling pathways that mediate this protection is an important next step for the field. Harnessing the protective role of NO and related signaling pathways could provide a therapeutic avenue that prevents synapse loss early in disease.

## 1. Alzheimer's Disease

Dementia is a form of neurodegenerative disorder, generally characterized by a disease specific loss of synapses and neurons which leads to memory impairment, cognitive decline, and eventually death [1]. Alzheimer's disease (AD) is the most common form of dementia, estimated to affect 36 million people worldwide, with this number predicted to triple by 2050 [2]. As the leading cause of disability and with the need for care in older people, the global economic cost associated with AD was estimated to be $604 billion in 2010 [3]. Currently, there is no known cure for AD, with available drugs only effective in mild to moderate cases and limited to treating the symptoms rather than the underlying cause of the disease [4]. As the world's population ages, AD will soon reach epidemic proportions; thus, there is an ever-increasing need for viable treatment options or a cure.

For the majority of AD cases, known as sporadic or late-onset AD, the precise etiology is currently unknown; however, a combination of advanced age and the inheritance of the *ε*4 allele of the apolipoprotein E gene can act as significant risk factors [5]. In the rare and inherited form of AD, known as familial or early-onset AD, several genetic mutations have been identified. The most common familial AD mutations occur in either the presenilin-1 or presenilin-2 genes (*PSEN1*,* PSEN2*), with duplications and mutations in the amyloid precursor protein (encoded by* APP*) also linked to the disease [6, 7]. The average age of onset for sporadic AD patients is between 65 and 80 years, while familial patients experience a drastically reduced age of onset, sometimes as early as the mid-20s.

The major neuropathological hallmarks of AD are the accumulation and aggregation of two proteins: *β*-amyloid (A*β*), in the form of extracellular plaques, and hyperphosphorylated tau, as intracellular neurofibrillary tangles [1, 8]. A pathogenic shift in the processing of the APP by two enzyme complexes, *β*-secretase and *γ*-secretase (of which the presenilins are catalytic subunits), results in the production of A*β* peptides [7]. These can form aggregates that disrupt cell signalling, trigger inflammatory immune responses, and cause oxidative stress [9]. When tau, a microtubule-associated protein, becomes hyperphosphorylated, it loses the ability to stabilise neuronal microtubules and abnormally accumulates in axons, dendrites, and cell bodies [10]. This disrupts vital transportation systems within the neuron and can trigger the activation of signaling pathways that lead to neuronal death [11]. A major problem in the field is that the models used to study AD provide only limited representations of this complex disease. The differences between rodent AD models and the human condition, coupled with a lack of clear understanding of disease progression, have contributed to the limitations of drugs in the clinic for AD.

## 2. Multifactorial Disease and the Failure of Drugs in the Clinic

AD is a complex and multifactorial disorder, which has made studying disease pathogenesis problematic. Studying snapshots of AD, through the window of postmortem tissue, has led to a complicated and at times uninterpretable mass of data. The key to understanding the disease must lie in engaging in longitudinal studies. Central to this has been the development of agents that can accurately image disease progression, through the analysis of biomarkers. Emerging data from long-term studies suggest that disease pathogenesis commences decades before cognitive decline [12, 13]. Oxidative and nitrosative stress, the result of increased levels of reactive oxygen and nitrogen species, respectively, have been reported in AD brains before the accumulation of A*β* and phosphorylated tau [14, 15]. The production of reactive oxygen and nitrogen species is both exacerbated by and can induce the formation of A*β* and phosphorylated tau [9]. In addition, disruptions to neuronal calcium signalling, mitochondrial dysfunction, and inflammation caused by the activation of microglia have all been reported to contribute to AD pathogenesis [16, 17]. Collectively, these pathogenic mechanisms result in synaptic loss and neuronal death, especially for cholinergic neurons found in the brain regions responsible for memory and language [18]. Ultimately, the disease spreads throughout the brain contributing to cognitive decline and eventually leading to death.

The complex pathogenesis of AD, coupled with the inaccessible nature of human brain tissue, has hindered the identification and development of prospective pharmaceuticals. During the period of 1998 to 2011, it is estimated that over 100 potential compounds targeting the treatment of AD have failed in the clinic, leaving only a handful of approved therapeutics addressing the cognitive symptoms but not the disease itself [19]. The primary pharmaceuticals currently available to AD patients are cholinesterase inhibitors (Donepezil, Rivastigmine, and Galantamine) and NMDA receptor antagonists (Memantine). These drugs have been shown to reduce memory loss and slow disease progression temporarily in some patients by 6–12 months [20]. With the development of imaging agents that can measure amyloid deposition, along with an improved knowledge of genetic risk factors, the possibilities for discerning the early events in disease pathogenesis are becoming a reality. Now it is essential that there is investment in longitudinal studies to investigate genetic contributions to disease processes in patients. In considering the development of effective drugs for AD, we need to identify early events that could afford protection to neurons and synapses. Recent findings suggest that one signaling molecule that warrants further investigation is nitric oxide.

## 3. Nitric Oxide in Alzheimer's Disease: Mechanisms and Effects

As a gasotransmitter that is freely diffusible across membranes, nitric oxide (NO) makes for a powerful signaling molecule with far-reaching cellular consequences that can be both protective and maladaptive. The multiple physiological effects of NO as a vasodilator, inflammatory mediator, and neuromodulator allow for a coordinated effect on brain function. NO is synthesized by three distinct genes,* NOS1*,* NOS2*, and* NOS3*, that encode the neuronal, inducible, and endothelial NO synthases (nNOS, iNOS, and eNOS), respectively. Both nNOS and eNOS are constitutively expressed, with their activation dependent on Ca^2+^/calmodulin, whilst iNOS expression is induced in inflammatory cells and is not dependent on Ca^2+^/calmodulin. Each of these cell types (neurons, endothelial cells, and inflammatory cells) is altered in Alzheimer's brain. There are deficits in the cerebrovasculature, characterized by the breakdown of the blood-brain barrier, as well as increased inflammatory signaling and alterations in neuronal signaling, all key components of AD [21]. Each of the three NOS isoforms has been postulated to play a role in either AD progression or prevention, leading to a seemingly conflicting message about the role of NO in AD and whether NO is neuroprotective or neurotoxic.

The signaling pathways of NO converge on three main cellular effects, all of which have been identified to play a role in AD: signaling via soluble guanylate cyclase and the cyclic guanosine monophosphate (cGMP) pathway [22]; direct S-nitrosylation of protein cysteine residues (addition of a nitrosyl ion NO^−^ to generate a nitrosothiol, RS-N=O) (reviewed in [23]); and protein tyrosine nitration (addition of nitrogen dioxide NO_2_ to generate 3-nitrotyrosine) [24]. Diversion of NO signaling towards one of these pathways over another depends on the local cellular microenvironment, including levels of transition metal complexes and redox status [25]. In addition, at high concentrations, NO reacts with superoxide anion that is formed as a by-product of respiration, to generate peroxynitrite (ONOO^−^), a highly reactive oxidant and cytotoxic species [26]. Thus, the production of peroxynitrite links high levels of NO release with oxidative stress. The numerous effects of NO in the multicellular environment of the brain have complicated the analysis of NO in the etiology of AD. Previous studies in postmortem tissue and animal models have yielded a complex proposition of NOS expression changes and NO signaling in AD (Table 1).

Synthesizing this data to assess a meaningful role for NO and NOS activity in AD is an impossible task due to the study of different brain regions and disease states, in addition to using different techniques and markers to quantitate NO. In the context of a multicellular environment, such as the brain, the source of NO (i.e., from which NOS enzyme, the signaling molecule, is derived) is important when considering normal physiological roles versus pathological effects. For example, iNOS releases higher levels of NO (up to the micromolar range), compared to nanomolar levels by eNOS or nNOS [27]. Because of its short half-life, there is a limited ability for NO to diffuse in three-dimensional space [28]. Thus, the source of NO directly affects its local concentration. Even in the case of a single neuron, events at the cell body do not necessarily translate to signaling at the synapse. New studies need to be carried out which can address the precise temporal and spatial NO signaling at the synapse and at extrasynaptic sites. This precise localization of NO most likely underlies the differential neuroprotective versus neurotoxic effects.

## 4. Neuroprotective versus Neurotoxic Effects of Nitric Oxide

An area of controversy in regard to the involvement of NO in AD pathogenesis is the extent to which the molecule is neuroprotective or neurotoxic [29–31]. Several studies have demonstrated that NO holds neuroprotective properties through its induction of the cGMP pathway [32–34]. This triggers vasodilation and consecutive increases in the cerebral blood supply to neurons, reducing the potential for oxidative stress, in addition to minimizing excess Ca^2+^ influx through inhibition of NMDA receptors at glutamatergic synapses [32–34].

Within the brain approximately 15% of the oxygen consumed is reduced in a one-electron transfer to superoxide, the main downstream component of oxidative stress. The ability of NO to easily cross local membranes thus allows it to react with free superoxide from cellular respiration [35]. The resultant peroxynitrite from the NO/superoxide pathway has been shown to induce lipid peroxidation, which can result in Ca^2+^ dysfunction, as well as functional alterations to proteins through S-nitrosylation of cysteine residues and nitration of tyrosine residues, both molecular markers of AD [36–38]. Further evidence suggests that the upregulation of constitutive NOS leads to the uncoupling of the enzyme, with the resultant formation of peroxynitrite overriding the neuroprotective cGMP pathway [34, 39]. Discrepancies could be due in part to the challenges of measuring NO and peroxynitrite concentrations in situ (half-life < 3 s and half-life < 1 s, resp.), which prevents a clear distinction of the formation of neurotoxic peroxynitrite at the expense of protective NO [26]. Developments in more precise nanotechnology based measurements for NO and peroxynitrite have helped to demonstrate that the hypothesized cytotoxic effects of NO in AD are only observed once NO has been converted to peroxynitrite [26, 40, 41]. Further developments in the accuracy of methods to measure NO and peroxynitrite are required to fully appreciate the roles of these signaling molecules in AD.

A primary activator of nitrosative stress in AD is the release of excess Ca^2+^ into the cytosol from the overstimulation of NMDA receptors, a concept known as excitotoxicity [29, 42]. Under physiological conditions, repetitive stimulation of NMDA receptors is considered to strengthen long-term potentiation (LTP), enhancing synaptic plasticity in neurons and the encoding of memory and learning [43]. However, prolonged, high intensity activation of extrasynaptic NMDA receptors triggers cell death pathways [44]. It has been demonstrated that nNOS is colocalized with NMDA receptors in the postsynaptic density and that after Ca^2+^ influx into postsynaptic neurons NO acts as a retrograde messenger providing a positive feedback mechanism to maintain glutamate release through the NMDA receptors, strengthening LTP [35, 45]. However, NO has also been found to inhibit NMDA receptors through cGMP induction [33]. A significant reduction in NMDA receptors in the hippocampus and cortex of postmortem AD brains has also been observed [46, 47]. The reduction in NMDA receptors is postulated to underlie the cognitive decline of AD, with the upregulation of NO a compensatory yet potentially neurotoxic mechanism to increase glutamate release in attempts to maintain LTP [48, 49]. Close associations of nNOS and NMDA receptors are central to this compensatory role of NO. Understanding the signaling pathways of synaptic versus extrasynaptic receptors is the next challenge for the field.

## 5. The Contribution of Nitrosative Stress to A***β*** and Tau Pathology

A rare consensus in the literature regarding NOS and NO in AD is that iNOS expression is increased in microglia and astrocytes during A*β* elicited inflammatory and immune responses [30, 50, 51]. This increased expression of iNOS in microglia and astrocytes generates elevated levels of NO and peroxynitrite through the NO/superoxide pathway, in addition to ROS and other neurotoxic molecules that can lead to neuronal death [50, 51]. Removal of iNOS in transgenic AD mice or the use of iNOS inhibitors to block NO production has been shown to protect against A*β* induced neurotoxicity, indicating that nitrosative stress may be one of the key factors mediating A*β* pathogenesis in AD [50, 52]. The involvement of NO in facilitating the neuropathogenesis of A*β* highlights the potentially significant role of NO in disease progression.

Increased NO synthesis due to overactivation of neuronal NMDA receptors and microglial activation, in combination with the properties that allow retrograde messenger activity, has implicated both intracellular and extracellular sources of pathogenic NO to neurons [31]. In aging rat hippocampal neurons nitrotyrosination of presenilin-1 caused it to increase A*β*1-42 production in a similar manner observed by* PSEN1* mutations associated with familial AD [37]. This finding points to a specific mechanism for how nitrosative stress could potentially induce one of the hallmarks of AD [37]. Furthermore, although the presence of S-nitrosylated proteins and tau has been demonstrated in AD, prolonged NO exposure can induce the formation of cytoplasmic tau oligomers in SH-SY5Y cells, providing evidence of a potential mechanism underlying tau neuropathogenesis in AD [53]. Experiments in cell lines using overexpression of AD markers have provided clues to mechanism but this research has a limited capacity to model AD effectively. Recapitulation of findings in clinically relevant samples is now essential. Induced pluripotent stem cells derived from sporadic Alzheimer's patients provide that opportunity.

## 6. Potassium Channels and Nitric Oxide Augmentation of Synaptic Plasticity and Neuronal Excitability

One of the major problems with the study of postmortem tissue is that the very cells required for study (i.e., vulnerable neurons) have been lost in the disease, which can lead to difficulties in assessing the changes identified between cases and controls. An important level of study in AD research is identifying early changes in neurons that may lead to degeneration or survival. The manipulation of these pathways may then provide potential targets for interventions.

Recent studies using AD mouse models have been used to identify early changes in neurons that could be targeted. The 3x Tg-AD mouse model bears mutations in three genes involved in familial AD:* APP*;* PSEN1*; and* MAPT*, encoding amyloid precursor protein; presenilin-1 (part of the *γ*-secretase complex); and tau. Consequently, these mice exhibit progressive neuropathology, including plaques and tangles, in addition to hippocampal synaptic dysfunction. An early characteristic of Alzheimer's brain is the loss of synapses and this occurs prior to memory loss. Synapse density therefore provides a better correlate with cognitive deficits than the classic hallmarks of plaques or tangles [54, 55]. A study of presymptomatic AD mice (i.e., before the development of cognitive behavioral changes) indicated that NO functions to maintain both LTP and long-term depression, while increasing the probability of neurotransmitter release [56]. In this way, NO signaling pathways are altered as a means to promote synaptic plasticity. Together these data suggest that NO increases the excitability of presynaptic neurons to promote neurotransmitter release in a pathologically dampened system. It remains unclear how NO mediates increased excitability of presynaptic neurons. However, the most likely mechanism is modulation of presynaptic ion channel activity. M-channels (Kv7 channels), as critical regulators of neuronal excitability, provide a possible candidate for this role. M-channels are voltage-gated outward potassium channels that remain open at the resting membrane potential of neurons. As such, increases in M-current reduce neuronal excitability, while M-current inhibition increases action potential firing. In sensory neurons NO is a potent neuromodulator with the ability to increase excitability by inhibiting M-current [57, 58]. NO-mediated changes in excitability have been identified in the mouse hippocampus, modulating outward potassium currents [31]. These effects included potentiation of Kv2 currents and suppression of Kv3 currents, which together promoted sustained action potential firing [31]. Indeed M-channel modulators are being touted as therapeutic possibilities for AD, amongst other neuronal excitability disorders [59]. Modifiers of potassium channel activity and neuronal excitability could therefore yield effective drug targets.

## 7. Nitric Oxide Suppression of Inflammatory Signaling

Other recent studies have also challenged the view that NO and proinflammatory factors drive disease progression in AD. Manipulating the effect of inducible NO in mice to levels equivalent to those in humans has led to some interesting results. These data suggest that local immune suppression, rather than immune activation, leads to degeneration of specific brain regions [60]. Elevated cerebrovascular NO levels increase NF*κ*B/p65 signaling in epithelial cells, preventing leukocyte trafficking [61]. The AD mouse model termed 5xFAD overexpresses mutant human* APP* with the Swedish (K670N, M671L), Florida (I716V), and London (V717I) mutations along with human* PSEN1* bearing two mutations (M146L and L286V). Consequently, in 5xFAD mice, scavenging NO boosts the trafficking capacity of epithelial cells and enhances recruitment of monocytes/macrophages into the brain from the periphery [61]. Consistent with this, systemic administration of the NO scavenger, rutin, reduces amyloid plaques in various mouse models of AD [62, 63], potentially via increased macrophage recruitment to the CNS and more rapid clearing of A*β*. In assessing the reasons why numerous AD drugs have failed in the clinic, first we have to address the extensive time difference in disease progression in humans compared to the mouse models in which the drugs were tested. Development of clinically representative models and the timing of interventions need to be further considered. As a field we need to establish universality in sampling selection and longitudinal studies that represent a timespan appropriate to disease processes (e.g., 20 years). Central to this is a commitment to long-term funding of such projects.

## 8. Summary

It is difficult to identify the specific contribution of NO and the extent to which NO synthases influence the development of AD. Human postmortem brain tissue has provided a snapshot of the final stages of the disease but has failed to represent early changes in the AD brain. Meanwhile, animal models have provided a more dynamic insight into disease pathogenesis but do not represent the complexities of the sporadic human disease. Future studies need to utilize human patient-derived neuronal cell models to elucidate the contribution of NO to neuroprotection and neurotoxicity in AD [64]. The development of inhibitors of the specific NOS isoforms for use in AD models will help to elucidate the neuroprotective and neurotoxic sources of NO. An emphasis on identifying the ionic mechanisms that cause alterations in NO-mediated excitability in AD may lead to the development of new drug targets.

New data suggest that alterations in NO signaling function as a compensatory mechanism to coordinate neuroprotective responses at the failing synapse and that the loss of local immune responses and amyloid clearance are likely more relevant to disease pathogenesis than increases in proinflammatory neurotoxic signaling. Identifying suitable NO or voltage-gated potassium channel modulatory drugs could therefore provide preventive action against synaptic loss and AD pathology.

## Figures and Tables

**Table 1:** Alterations in the expression and activity of nitric oxide synthase (NOS) enzymes in Alzheimer's disease (AD) tissue and animal models.

Author	Methods	Tissue type/control	Results
Hyman et al., 1992 [65]	Immunocytochemistry staining of NOS in neurons using rabbit polyclonal antibody and peroxidase linked secondary antibody.	Hippocampus and temporal neocortex from AD and control postmortem brains. AD mean age: 80.85 ± 2.0 years. Control mean age: 60.4 ± 5.9 years. Five control subjects had brain abnormalities upon postmortem examination.	No significant difference between the expressions of NOS in AD neurons in comparison to controls.

Dorheim et al., 1994 [66]	L-Citrulline (coproduct of NO) was used as a marker of NOS activity in microvessels.	Brain microvessels from AD and control patients.	Significant increase in NOS activity in AD brain microvessels.

Benzing and Mufson, 1995 [67]	Nicotinamide adenine dinucleotide phosphate-diaphorase (NADPH-d) used as a marker for NOS in neurons.	AD and control postmortem brains (age, sex, brain weight, and postmortem interval matched).	Significantly higher levels of NADPH expression in AD neurons in comparison to controls.

Norris et al., 1996 [68]	mRNA expression levels of nNOS and NADPH-d staining used as markers for nNOS expression.	Frontal cortex, visual cortex, and hippocampus of AD and control postmortem brains.	A decrease (not significant) in cellular abundance of nNOS in AD brains in comparison to controls. A significant decrease in the number of cells expressing nNOS in distinct brain regions.

Gargiulo et al., 2000 [69]	Immunohistochemistry staining with monoclonal antibodies for NOS and protein kinase C (PKC) expression.	Regions of the temporalis gyrus from AD and control postmortem brains.	A significant decrease in NOS levels from AD brains, no change in PKC expression levels.

Lüth et al., 2001 [70]	Immunohistochemistry and western blotting of iNOS and eNOS expression levels in three tissue types.	Sporadic AD postmortem brains, human APP transgenic mice, and electrolytic cortical lesions in rat tissue. Age and postmortem interval matched for human controls. Aged and nontransgenic mice matched controls.	Increased expression of iNOS and eNOS in both human AD and transgenic mice reactive astrocytes in comparison to controls.

Venturini et al., 2002 [71]	Optical, fluorescence, and NMR spectroscopy was used to determine A*β*_25–35_ interaction with NADPH-d and downstream effects on NOS activity.	Neuronal and glioma-like rat cell lines and appropriate controls.	A*β*_23–35_ interacts with NADPH-d, decreasing the availability of the substrate for cNOS and strongly reducing cNOS activity.

Stepanichev et al., 2008 [72]	NADPH-d histo- and immunocytochemistry used as a marker for nNOS and iNOS expression.	Cerebral and hippocampus A*β*_25–35_ administered rat tissue and non-A*β* rat tissue as controls.	A*β*_25–35_ did not influence nNOS or iNOS mRNA or protein expression. A*β*_25–35_ increased nNOS activity but not iNOS.
